# A locally funded Puerto Rican parrot (*Amazona vittata*) genome sequencing project increases avian data and advances young researcher education

**DOI:** 10.1186/2047-217X-1-14

**Published:** 2012-09-28

**Authors:** Taras K Oleksyk, Jean-Francois Pombert, Daniel Siu, Anyimilehidi Mazo-Vargas, Brian Ramos, Wilfried Guiblet, Yashira Afanador, Christina T Ruiz-Rodriguez, Michael L Nickerson, David M Logue, Michael Dean, Luis Figueroa, Ricardo Valentin, Juan-Carlos Martinez-Cruzado

**Affiliations:** 1 University of Puerto Rico at Mayagüez, Mayagüez, Puerto Rico; 2 University of British Columbia, Vancouver, BC, Canada; 3 Axeq Technologies, Seoul, South Korea; 4 Cancer and Inflammation Program, National Cancer Institute, NIH, Frederick, MD, USA; 5 Compañía de Parques Nacionales de Puerto Rico, San Juan, Puerto Rico; 6 Department of Natural and Environmental Resources, San Juan, Puerto Rico

**Keywords:** *Amazona vittata*, Puerto rican parrot, Genome sequence, Annotation, Assembly, Local funding, Education

## Abstract

**Background:**

*Amazona vittata* is a critically endangered Puerto Rican endemic bird, the only surviving native parrot species in the United States territory, and the first parrot in the large Neotropical genus *Amazona*, to be studied on a genomic scale.

**Findings:**

In a unique community-based funded project, DNA from an *A. vittata* female was sequenced using a HiSeq Illumina platform, resulting in a total of ~42.5 billion nucleotide bases. This provided approximately 26.89x average coverage depth at the completion of this funding phase. Filtering followed by assembly resulted in 259,423 contigs (N50 = 6,983 bp, longest = 75,003 bp), which was further scaffolded into 148,255 fragments (N50 = 19,470, longest = 206,462 bp). This provided ~76% coverage of the genome based on an estimated size of 1.58 Gb. The assembled scaffolds allowed basic genomic annotation and comparative analyses with other available avian whole-genome sequences.

**Conclusions:**

The current data represents the first genomic information from and work carried out with a unique source of funding. This analysis further provides a means for directed training of young researchers in genetic and bioinformatics analyses and will facilitate progress towards a full assembly and annotation of the Puerto Rican parrot genome. It also adds extensive genomic data to a new branch of the avian tree, making it useful for comparative analyses with other avian species. Ultimately, the knowledge acquired from these data will contribute to an improved understanding of the overall population health of this species and aid in ongoing and future conservation efforts.

## Data description

A locally funded genomic sequencing project provided the first phase of genome sequencing of the Puerto Rican Parrot (*Amazona vittata*) (see Developing of the Local Community Involvement in Additional file 1). DNA was purified from a female *A. vittata* blood sample (see Additional file 2: Table S1), and sequencing was initiated with the construction of two genome libraries: the majority of sequencing used a short fragment library (~300 bp inserts), and scaffolds were generated using a long fragment library (~2.5 kb inserts). Raw Illumina HiSeq reads were processed and filtered using the Genome Analyzer Pipeline software (as per the manufacturer’s instructions at default parameters). Of the 309,060,168 paired-end reads and the 180,079,956 mate-pair reads, respectively, 86.48% and 85.14% passed QC, using the condition that if one read from a pair failed the QC, the entire pair was filtered out. Based on the total number of base pairs generated (see Additional file 3: Table S2), and the predicted genome size of 1.58 Gb [1], we calculated a total genome coverage of 26.89x depth: with 17.08x coverage for short fragment reads, and 9.8x for mate pairs (Table 1 and Additional file 3: Table S2) (see Sample Collection and Genome Sequencing sin Additional file 1). 

**Table 1:** **Average coverage of the Puerto Rican parrot genome in the current study based on the predicted genome size of 1.58Gb [**[1]**]**

**Sample**	**Sequence information**	**Total bases**	**Read count**	**Coverage**	**Total**
Pa9a	Pa9a_1	13,496,744,938	133,631,138		
(~300 bp inserts)	Pa9a_2	13,496,744,938	133,631,138	17.08X	
Pa9a	Pa9a-MP_1	7,743,004,915	76,663,415		
(~2.5 kbp inserts)	Pa9a-MP_2	7,743,004,915	76,663,415	9.90X	**26.89X**

We carried out two separate *de novo* assemblies, using Ray [2] software (Table 2) and SOAP*denovo*[3] (Additional file 4: Table S3), and selected the Ray assembly for use in all further analyses. Our genome coverage was approximately 76%, which, given some of the scaffolds may be overlapping and could not be properly assembled, might be slightly overestimated. (see Assembly in Additional file 1). We evaluated assembly by comparing the entire collection of transcripts listed for *G. gallus* in the NCBI Entrez Gene database using local BLAST [4] and found that > 70% of the chicken transcripts were present, and as much as 11% of scaffolds shared similarity with at least one *G. gallus* sequence at average density of 1.39 genes/kbp (Table 3; Additional file 5: Figure S1). 

**Table 2:** **Results of the genome assembly by Ray [**[2]**]**

	**Category**	**≥ 100 nt**	**≥ 500 nt**
**Contigs**	Number	358,398	259,423
	Total length	1,137,438,369	1,116,807,713
	Average	3,173	4,304
	Largest	75,003	75,003
	Median	1,637	2,774
	**N50**	**6,841**	**6,983**
**Scaffolds**	Number	245,947	148,255
	Total length	1,184,594,388	1,164,566,833
	Average	4,816	7,855
	Largest	206,462	206,462
	Median	1,048	2,913
	**N50**	**19,033**	**19,470**

**Table 3:** Annotation summary

**Scaffolds mapped to:**	**Scaffolds**		**mRNAs**^**+**^			**Repeats**		
	**N**	**(%)**^**#**^	**N**	(%)*	**% of the scaffold**	**N**	(%)*	**% of the scaffold**
*G. gallus* genome only	53,345	22%	1,256	5%	8%	88,157	76%	7.7%
Unmapped	105,030	43%	1,429	2%	22%	125,470	48%	19.4%
*T. guttata* genome only	26,078	11%	4,206	21%	7%	87,592	93%	2.1%
Mismatched	54,621	22%	12,030	27%	2%	266,478	98%	1.0%
*G. gallus* and T. *guttata*	6,873	3%	1,426	26%	3%	32,994	98%	1.2%
**Total**	**245,947**	**100%**	**20,347**	**11%**	**4%**	**600,691**	**59%**	**4.3%**

^+^ mRNAs are from *G. gallus.*

# Percentage values are from total number of scaffolds.

* Percentage values are from the number of scaffolds in that category.

RepeatMasker software (http://www.repeatmasker.org) was used to search scaffolds for the presence of the known repeat classes with known repeats found on 59% of the scaffolds (see Annotation in Additional file 1). In addition, we used manual annotation, both by annotation scaffolds for gene and repeat elements and by annotating known genes, to validate high-throughput annotation, and using this, we designed and carried out a student development program (see Genome Annotation and Education in Additional file 1).

Comparative analyses of the *A. vittata* scaffolds against the chicken (*Gallus gallus*) [5] and zebra finch (*Taeniopygia guttata*) [6] genomes using local BLAST [4] resulted in 93.4 Mbp of total length of alignments to the chicken genome with 82.7% identity on average (average bit score 577.3), and 41.7 Mbp of total length of alignments to the zebra finch genome with 84.5% identity on average (average bit score 431.1).

**Figure 1:**
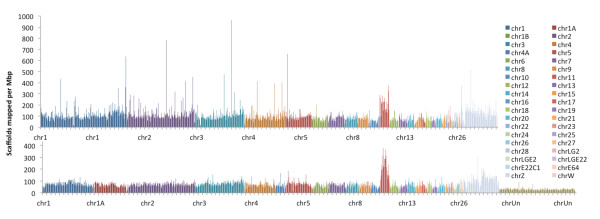
**Density of the*A. vittata*scaffolds that shared similarity with fragments of chicken and zebra finch genomes (Top) Chicken (*G. gallus*genome (per Mbp) and (Bottom) zebra finch (*T. guttata*) genome (per Mbp).** Different chromosomes are represented by different colors as shown in the legend on the right. Chromosomal locations, lengths and quality of alignments to the two genomes by BLAST are presented in Additional file 6: Table S4.

**Figure 2:**
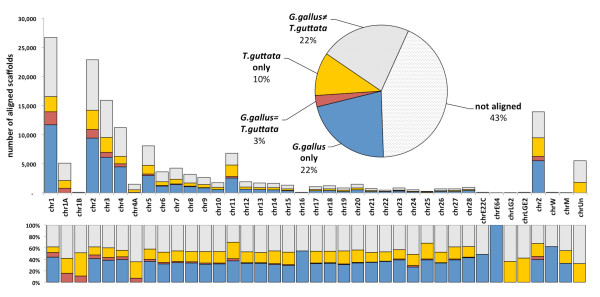
**Proportion of sequences with some similarity across the two avian genomes (*G. gallus*and*T. guttata*).***A. vittata* scaffolds are classified into five categories (**A**) *unmapped* - those that were not found any similar sequence, (**B**) *chicken only* – those that shared similarity only with a fragment of *G. gallus* genome; (**C**) *finch only* – those that shared similarity only with a *T. guttata* genome; (**D**) *mismatched* – those scaffolds that shared similarity with sequences of *G. gallus* and *T. guttata* genomes but mapped to different chromosomes in the two species; (**E**) *matched* – those that mapped to the same chromosome in reference genomes of the two avian species. Proportions are represented as totals **(pie chart),** absolute numbers **(top)** and proportions per chromosome **(bottom)**. The associated data are provided in Additional file 9: Table S5.

The top BLAST alignments were sorted by the average of their locations, and their frequencies were calculated in 1 Mbp bins and plotted along all of the chromosomes for both *G. gallus* and *T. guttata* genomes using Circos [7] (Figure 1). The chicken genome coverage was higher (109 scaffolds per Mbp in chicken on average vs. 72 in zebra finch), but the chicken genome also had more locations with higher genome coverage. As high as 57% of the scaffolds could be partially aligned to one or both of the genomes: 21.7% aligned only to *G. gallus*, and 10.6% aligned exclusively to *T. guttata*, while 25% aligned to both genomes (Figure 2). These data are presented and summarized for chicken in Additional file 6: Table S4.A, for zebra finch in Additional file 7: Table S4.B, and the complete information in Additional file 8: Table S4.C. 

**Figure 3:**
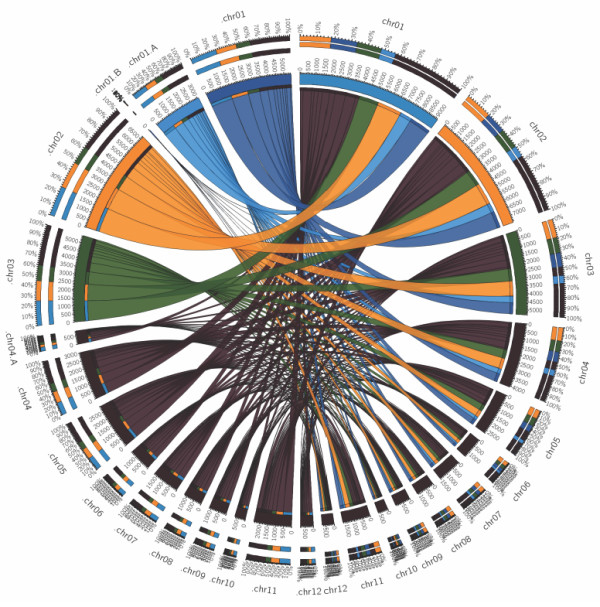
**Synteny of alignment of the*A. vittata*scaffolds to two avian reference genomes (*G. gallus*and*T. guttata*).** The connecting lines show the proportion of scaffolds that mapped to *T. guttata* chromosomes on the left side to *G. gallus* chromosomes on the right side. The chromosomes are shown in order from top to bottom and designated in the same color for the both species. For simplicity, different colors are used only for the three largest chromosomes. Chromosome 1 in *G. gallus* corresponds to chromosomes 1, 1A and 1B in *T. guttata* shown in different shades of blue.

Although a large proportion of scaffolds shared some similarity with the two avian genomes, there was also discordance as only 12.6% of the scaffolds (2.8% of the total number of scaffolds) aligned to the same chromosome in both species (Figure 2, top and Additional file 9: Table S5), and the proportion of discordance varied across chromosomes, with the lowest value on chromosome 11 (Figure 2, bottom and Additional file 9: Table S5). While this lack of synteny could point to extensive rearrangements during the evolutionary history, the proportions of scaffolds discordantly aligned between chromosomes seemed to be distributed similarly relative to chromosome lengths, indicating a significant random component (Figure 3). To test this, we selected the 200 longest scaffolds and independently queried 500 bp ends to the chicken genome. Of these, only 10 scaffolds (5%) showed discordance by aligning to the opposite ends to two or more different chicken chromosomes (see Comparative Analysis in Additional file 1).

In summary, these data represent the first assembly of a genome sequence for a parrot endemic to the United States, and also the first genome of a species from the diverse and ecologically important genus, *Amazona,* native to South America and the Caribbean. The assembled sequence provides a starting point towards completing and annotating a draft genome sequence. The data available at this coverage will be helpful in designing the future sequencing efforts, and can also be used for annotation and comparative genomic studies across the growing amount of avian genome data [5,6,8], which is essential given the growing rate of extinction among avian species worldwide.

## Availability of supporting data

The raw reads are available at the ENA (accession #PRJEB225). Scaffolds and the assembly parameters have been submitted to the GenBank (accession #PRJNA171587), and all data, including FASTA files of contigs, scaffolds, corresponding assembly parameters, and annotation data are available in *Giga*DB [9]. The links to all the supplementary tables and databases are listed in (Additional files 2, 3, 4, 6, 7, 8, 9, 10, 11, 12, 13, 14, 15, and 16) and can also be accessed at http://genomes.uprm.edu/gigascience/Supplementary Tables/.

## Competing interests

Oleksyk TK, Pombert JF, Mazo A, Ramos B, Guiblet W, Afanador Y, Ruiz-Rodriguez CT, Nickerson ML, Logue D, Dean M, Figueroa L, Valentin R, and Martinez-Cruzado JC do not have competing interests. Siu D is employed by Axeq Technologies; the company which carried out the DNA Sequencing.

## Authors' contributions

TKO, LF, RV, MD, MLN, DL and JCMC came up with the idea, and designed the experiments. TKO, WG, YA, CTRR and JCMC organized public support and raised the funds. TKO, AMV, BR, YA, CTRR and RV collected, extracted and quantified DNA. DS performed sequencing and assembly by SOAPdenovo. JFP performed assembly by Ray. TKO and WG designed the data browser webpage. TKO, JFP, MLN, DL, MD and JCMC wrote the paper. All authors read and approved the final manuscript.

## Note from the editors

A related commentary by Stephen O’Brien on the issues surrounding this work is published alongside this article [10].

## Supplementary Material

Additional file 1 Supplementary materials. Click here for file

Additional file 2 **Table S1.** Quality and volume of four DNA samples extracted from whole blood of two *Amazona vittata* parrots selected for the genome sequencing. Click here for file

Additional file 3 **Table S2.** Results of the genome sequencing (Illumina HiSeq, Axeq Technologies). Pa9a_1 and Pa9a_2 represent the opposite ends of the 300 bp short reads, and the Pa9a-MP_1 and Pa9a-MP_2 are the 2,500 bp mate pairs (MP). All sequences were 101 bp long. Click here for file

Additional file 4 **Table S3.** Results of the genome assembly by SOAPdenovo [8]. Click here for file

Additional file 5 **Supplementary figures. Figure S1.** Venn diagram of the overlap between the number of A. vittata scaffolds and the G. gallus transcripts from GenBank that were mapped to them by BLAST. **Figure S2.** A single example of chimera detected on scaffold-74754 after visual inspection of reads mapped to 100 largest scaffolds. **Figure S3.** Percentage of scaffolds containing fragments with > 95% similarity to GenBank sequences. **Figure S4.** Comparison between categories of A. guttata scaffolds (described earlier in Figure 2): The box plots show the medians, Q1, Q3 and the extreme values. The means are shown in Table 3. A. Distribution of scaffold lengths; B. Distribution of densities of genes mapped per kbp of scaffold length. C. Differences in the distribution of proportion of the length of the scaffold mapped to a G. gallus transcript from NCBI Entrez Gene database. D. Differences in the distribution of proportion of the length of the scaffold mapped to a known repeat class using RepeatMasker software [5]. **Figure S5.** Distribution of major classes of repetitive sequences found on A. vittata scaffolds. **Figure S6.** Relationship between the quality scores of the alignments between the parrot scaffolds to the chicken and zebra finch genomes: A. All scaffolds. B. Mismatched scaffolds only (those scaffolds that shared similarity with sequences of G. gallus and T. guttata genomes but mapped to different chromosomes in the two species; see classification in Figure 2). C. Matched sequences only (those that mapped to the same chromosome in reference genomes of the two avian species). **Figure S7.** Relationship between the size of a scaffold and the quality of its alignment to T. guttata and/or G. gallus genome sequence: A. All scaffolds aligned to the T. guttata genome. B. All scaffolds aligned to the G. gallus genome. C. Scaffolds from T. guttata that Mismatched scaffolds mapped to different chromosomes in G. gallus; see classification in Figure 2). D. Scaffolds from G. gallus that Mismatched scaffolds mapped to different chromosomes in T. guttata). E. Matched sequences from T. guttata only (those that mapped to the same chromosome in reference genomes of the two avian species), F. Matched sequences from G. gallus only (those that mapped to the same chromosome in reference genomes of the two avian species). **Figure S8.** Small fragments are repeat- rich and gene-rich: A. Relationship between the length of the scaffolds and the proportion of it length matched to the G. gallus sequences from NCBI Entrez Gene database. B. Relationship between the length of the scaffolds and the proportion of it length designated by RepeatMasker as repetitive sequence. Click here for file

Additional file 6 **Table S4A.** Summary of the alignment of *A. vittata* sequences to the *G. gallus* genome sequence containing only the top alignment for each scaffold, its chromosomal position and quality scores. Click here for file

Additional file 7 **Table S4B.** Summary of the alignment of *A. vittata* sequences to the *T. guttata* genome sequence containing only the top alignment for each scaffold, its chromosomal position and quality scores. Click here for file

Additional file 8 **Table S4C.** The database of the alignment information of *A. vittata* sequences to *G. gallus* and *T. guttata* genome sequence by BLAST. Click here for file

Additional file 9 **Table S5.**Proportions of sequences with some similarity that mapped to chromosomes of two reference avian genomes*(G. gallus and T. guttata).* Click here for file

Additional file 10 **Table S6A.The summary of the database of GenBank sequences with more than 95% similarity with the parrot scaffolds.** Click here for file

Additional file 11 **Table S6B.** The database of GenBank sequences with more than 95% similarity with the parrot scaffolds found by BLAST. S7A. A map of *G. gallus* transcripts from NCBI Entrez Gene database that mapped to one of the *A. guttata* scaffolds. Click here for file

Additional file 12 **Table S7A.** A map of *G. gallus* transcripts from NCBI Entrez Gene. Click here for file

Additional file 13 **Table S7B.** The database of alignments between of *G. gallus* transcripts from NCBI Entrez Gene database and *A. guttata* scaffolds by BLAST. Click here for file

Additional file 14 **Table S8.** Distribution of different cases of repetitive elements among different classes of *A. guttata* scaffolds. Click here for file

Additional file 15 **Table S9.** Bioinformatics tools and outputs for scaffold and gene annotation. Click here for file

Additional file 16 **Table S10.** An example of annotation output produced by a student in the Genome annotation class using *A. vittata* genome. Click here for file
