# Supplementary material for: A locally funded Puerto Rican parrot (Amazona vittata) genome sequencing project increases avian data and advances young researcher education
Source: Gigascience. 2012 Sep 28;1:14. doi: 10.1186/2047-217X-1-14 (PMC3626513; doi:10.1186/2047-217X-1-14)
Supplement: Additional file 1 — Supplementary materials. [file 2047-217X-1-14-S1.doc]

**Supplementary materials**

**Background**

The Puerto Rican parrot (*Amazona vittat*a) is the only surviving native parrot species in the United States [1]. This endangered bird was once abundant throughout the island of Puerto Rico, but the population declined drastically during the 19th century with the decimation of the old growth forest habitat [2]. One of the first people to call attention to the need for its conservation was President Theodore Roosevelt, who formed the Luquillo Forest Reserve for the purpose of protecting the parrots’ nesting grounds [1]. El Yunque National Forest and a few other remote locations were spared from deforestation, allowing a small population of parrots to survive [2-4]. At the end of the 20th century, the species came very close to extinction: only 16 birds remained alive in 1975. The Puerto Rican parrot has been included on the Endangered Species List since 1967, and listed as critically endangered by the World Conservation Union since 1994. A recovery effort has been initiated by two captive breeding programs, one at the Luquillo Aviary close to El Yunque and another at the Rio Abajo State Forest. This effort has proven to be a success; the captive population is steadily increasing, and several captive-bred birds have been released into the wild.

Genome-wide consequences of the population decline and their effects on the species recovery have never been assessed. Similarly to most birds, the *A. vittata* genome is relatively small (1.58 Gb) – less than half the size of the human genome [5]. It is also highly invariable due to the recent population bottleneck [6]. These two aspects made the Puerto Rican parrot an ideal candidate for genome sequencing and assembly: the small genome size allows high sequencing coverage, while the low amount of genetic variation permits accurate alignments between sequenced fragments resulting in fewer assembly errors.

**Methods**

**Development of the local community involvement**

The Puerto Rico National Parks Company recently started exhibiting Puerto Rican parrots and related species at the Puerto Rico Zoo Juan A. Rivero. This exhibition will bring attention to the conservation effort for this endangered species. In conjunction with the comparative genomics study to be conducted at the University of Puerto Rico, the parrot exhibition will become a valuable research and educational resource for the local and international scientific community. The sequence of the Puerto Rican parrot will be used as a reference to study differences between species and to find genomic regions responsible for adaptation and survival.

**Sample collection and genome sequencing**

All sampling procedures were carried out as approved by the University of Puerto Rico at Mayaguez Institutional Animal Care and Use Committee (IACUC#201109.1), and in accordance with the guidance for the Endangered Species Act. A veterinarian designated by the Puerto Rico Department of Natural and Environmental Resources (DNER) collected blood from two non-reproductive captive female birds at the Rio Abajo aviary during a routine health check procedure. Syringes were heparinized before samples were taken. Approximately 1.5 cc of blood was extracted from each bird using a 3 cc syringe with a 27-gauge needle. Birds were placed into their cages after the procedure and remained under observation for the rest of the day to make sure that no harm had been done. Samples were aliquoted and extracted multiple times (23 times for one bird and 3 times for the other) to produce a total of 2 ug of purified DNA with the QIAamp® DNA Blood Midi kit (Qiagen, Venlo, The Netherlands) according to the manufacturer's instructions and using the following modifications. Blood samples were diluted in 1x PBS in a 1:10 ratio. The samples were incubated at 70ºC in the lysis buffer and protease for 2–12 hours. After cellular lysis, DNA was precipitated with 100% ethanol and the solution was passed twice through the Midi column. The DNA was eluded two times with 300 µl of pre-warmed elution buffer, incubated for 10 minutes at room temperature and then centrifuged for two minutes. After the DNA isolation, DNA purity was assessed with a BioTek® microplate reader (BioTek, Winooski, VT, USA (A260/A280) compared to a blank control after correcting for the baseline (A320). The remainder of the tissue material has been refrigerated at -80**°**C. The concentrations and quality of DNA were re-assessed using a fluorometer and the Quant-iT™ PicoGreen® ds DNA binding assay (Invitrogen, Carlsbad, CA, USA from which four of the best DNA samples were chosen. These samples were shipped to Axeq Technologies (Rockville, MD, USA; and Seoul, South Korea) to be used for next generation sequencing, and the DNA concentrations were validated once more upon arrival (Table S1). The DNA concentrations did not deviate between different methods of measurement. One sample (Pa9a) has been finally selected and sequenced on Illumina HiSeq 2000 platform (Illumina, San Diego, CA, USA) with both fragment and paired-end sequencing approaches, resulting in a total of 42,479,499,706 bases (Table S2). The sequencing was initiated with the construction of two genome libraries: a short fragment library (~300 bp inserts) for sequencing the majority of the genome, and a long fragment library (~2.5 kb inserts) to generate scaffolds to be used to order and assemble contigs derived from the short fragment library. Raw Illumina HiSeq reads were processed and filtered using the Genome Analyzer Pipeline software provided by the manufacturer set to the default parameters. As much as 86.48% of the 309,060,168 paired-end reads, and 85.14% of the 180,079,956 mate-pair reads generated passed Illumina quality control (QC). If one read from a pair failed the QC, the whole pair was filtered out.

**Assembly**

We did two different *de novo* assemblies: we used Ray [7] software on 256 CPUs for 8 hours for the first assembly, and SOAPdenovo [8] for the second (Table S3). Ultimately, only the Ray assembly was used in this study, but the contigs and scaffold FASTA files for both assemblies and the associated parameter files have been deposited to our locally managed genome database [9] and in the *GigaScience* database [10].

For Assembly 1 with Ray: The Illumina paired-end and mate-pair reads were assembled together, with the *k*-mer defined iteratively. Here, we cautiously selected a *k*-mer value of 31 to ensure that the N50 parameter was not over-optimized (i.e., over-optimizing N50s may lead to chimeric contigs [11], as these values are indicative of the length of the contigs, but do not yield any information on their veracity). Furthermore, to assess the overall quality of the assembly, reads were subsequently re-mapped to the 10 largest scaffolds in order to detect regions harboring unusually high/low coverage, and potential assembly errors were manually reviewed. Of these, only one (scaffold 74754) contained a single chimera (Figure S2), while the other nine did not (i.e*.*, 1 error per 1,930,389 bp).

To test for the possibility of bacterial contamination, we queried all scaffolds against the entire GenBank nucleotide database [12], and filtered matches with more than 95% identity. Most of the scaffolds did not match any of the fragments in the database (87%). The rest of these seem to belong to sequences from either avian (6%) or mammalian (5%) DNA (Figure S3 and Table S6A). This indicates that contamination, if it exists, is most likely minimal. The database resulting from this query contains highly conserved elements and can be used for the subsequent annotation effort (Table S6B). In total, given that the genome size is predicted to be 1.58 Gb, with the total scaffold length of 1,184, 594,388 bp, we infer the overall coverage of the genome to be around 76%, a value that might be slightly overestimated given that some of the scaffolds may be overlapping but could not be assembled.

Sequence data was manipulated with custom Python scripts, and with the MUSCLE algorithm [13,14] within Geneious [15] that has been used for the local alignment. Local BLAST and BLAT were used for the similarity search. All statistical analyses in this study were performed with SAS 9.2 (1996-2012) software (SAS Institute, Cary NC, USA).

**Annotation**

To evaluate the current assembly, we compared the entire collection of transcripts listed for *G. gallus* in the NCBI Entrez Gene database using local BLAST [16]. Among the 28,846 queries, 20,138 (70%) have been found on 245,947 scaffolds, resulting in 27,431 matches (Figure S1 and Table 3). As much as 11% percent of scaffolds shared similarity with at least one *G. gallus* sequence at average density of 1.39 genes per kbp. The smallest number of matches was found on unmapped scaffolds (2%), but they were found there to be at the highest density (3.72 3 genes per kbp). Mapped Entrez Gene sequences made up 4% of the scaffold length on average, but in the scaffolds that were not mapped to either of the two avian genomes, the proportion was much higher (22%) (Table 3). While the unmapped scaffolds were the shortest (Figure S4A), they contained the highest density and percentage of the gene sequences (Figure S4B and C; Table S4A).

A database containing *G. gallus* gene sequences that were mapped to the parrot scaffolds and their locations is available from Table S7A and B. We used RepeatMasker software [17] to search scaffolds for the presence of the known repeat classes. Overall, 59% of the scaffolds contained at least one repeat, and the proportion was much higher in those that matched to another genome (Table 3). Even though a relative proportion of unmatched category of sequences that contained repeats was smaller than that in the other four categories, the percentage of its length classified as a repetitive sequence was much higher than in other classes (22%) (Figure S4D). The most common class of repeats found on scaffolds were the LINES/SINES, low complexity regions, and simple repeats, as they add up to 96% of the length of all repetitive sequences we found (Figure S5). A representative database of different repetitive elements and their locations on scaffolds is also hosted as supplementary information from our homepage and the *GigaScience* database [10]. There were no observable differences in the distribution of different cases of repetitive elements among different classes of scaffolds listed in Table 3 and Table S8).

**Genome annotation and education**

In addition to the preliminary annotation analysis, and with the goal of using the current genome sequence as an educational tool for training the next five generations of genomics and bioinformatics students at the University Puerto Rico at Mayaguez, we developed a strategy of manual annotation of the genome. Manual annotation is also used as a method to validate high-throughput annotation, and to canalize the desire of the local community to contribute to the project. Two annotation strategies are used: (1) annotation of scaffolds for gene and repeat elements, and (2) annotating known genes from other species.

According to the first strategy, each student in the Genome Annotation class (20 Undergraduate students) is given five from the list of 100 longest scaffolds ranging from 120 to 206 kbp in length and learns to apply a variety of bioinformatics tools (Table S9). Students divide each scaffold into 25 kbp segments and use an online BLAT tool to search against the chicken genome in the UCSC Genome Browser. When one segment would not align to the same chromosome as the other segments in the scaffold, the result is confirmed using an online UCSC BLAT query [18] against the zebra finch genome, as well as by the NCBI nucleotide BLAST [16] query against the chicken genome. As an output for each scaffold, a student receives an additive score of its segments, the leftmost and rightmost coordinates of the matches, the partial or complete RefSeq genes, and the number of conserved elements in at least four of the following six vertebrate species: human, mouse, rat, opossum, *Xenopus tropicalis*, and the zebrafish. To investigate the level of evolutionary conservation of each RefSeq gene, students also score the taxonomic groups where orthologs in NCBI’s Homologene or Gene are found. In addition, they look for the gene ontology in UniProtKB, always scoring the major biological processes in which the gene is involved, when known. Finally, students use an online version of RepeatMasker to score the number, type and extension of repetitive elements in their scaffolds. They also store the RepeatMasker detailed outputs with the coordinates and identification of each repetitive element. An example of the annotation output produced by a student is presented in Table S10.

The other strategy of annotation is to search for the genes known from other species in the assembled scaffolds. Students in the Genome Annotation class receive either one or two complete genes, depending on gene structure complexity. To annotate the coordinates where coding regions start and end, as well as those of the splice sites within coding regions, students first identify these sites in the chicken genome from Ensembl, and then localize them in the parrot genome using BLASTx against the chicken proteins and identifying the results of this query in the UCSC Genome Browser. All isoforms identified in UniprotKB for each gene are annotated.

**Comparative analysis**

Scaffolds were compared to the chicken (*Gallus gallus*) [19] and zebra finch (*Taeniopygia guttata*) [20] genomes using local BLAST [16]. First, the entire database of *A. vittata* scaffolds was queried against both reference avian genomes to assess coverage, mapping concordance and the long-range contiguity of the constructed scaffolds. Only the top alignment was taken into consideration for each scaffold. The alignment with two other avian genomes (*G. gallus* and *T. guttata*) resulted in 93.4 Mbp of total length of alignments to the chicken genome with 82.7% identity on average (average bit score 577.3), and 41.7 Mbp of total length of alignments to the zebra finch genome with 84.5% identity on average (average bit score 431.1). Despite the overall better alignment, gap lengths in chicken were much larger (43 vs. 16 per scaffold alignment in zebra finch genome). There was no relationship between the quality scores of alignment of the parrot scaffolds to chicken and zebra finch genomes (Figure S6A), but there was a positive correlation between the length of the scaffolds and the alignment score in zebra finch, which was not observed for the chicken scaffolds (Figure S6B). However, the longer scaffolds have better alignment scores in *T. guttata*, but not in *G. gallus* (Figure S7). On average, chicken genes constitute 4% of our scaffolds, and as much as 20% in the shorter unmatched fragments (Table 3). This may be the consequence of a simple tendency that gene rich-regions are the easiest to assemble: longer scaffolds contain less repetitive sequence (Figure S8.A), matched to more of these coding sequences (Figure S8.B).

**References**

1. Brinkley D: **The wilderness warrior: Theodore Roosevelt and the crusade for America**, 1st edn. New York: HarperCollins; 2009.
2. Snyder NFR, Wiley JW, Kepler CB: **The parrots of luquillo, natural history and conservation of the Puerto Rican parrot** Los Angeles; 1987.
3. Waide RB: **The Effect of Hurricane Hugo on Bird Populations in the Luquillo Experimental Forest, Puerto Rico**. *Biotropica* 1991, **23**(4):475-480
4. Brash AR: **The history of avian extinction and forest conversion on Puerto Rico**. *Biological Conservation* 1987, **39**(2):97-111.
5. Tiersch TR, Wachtel SS: **On the evolution of genome size of birds**. *The Journal of* 11 *heredity* 1991, **82**(5):363-368.
6. Brock MK, White BN: **Application of DNA fingerprinting to the recovery program of the endangered Puerto Rican parrot**. *Proceedings of the National Academy of Sciences* 1992, **89**(23):11121-11125.
7. Boisvert S, Laviolette F, Corbeil J: **Ray: simultaneous assembly of reads from a mix of high-throughput sequencing technologies**. *Journal of computational biology: a journal of computational molecular cell biology* 2010, **17**(11):1519-1533.
8. Li R, Zhu H, Ruan J, Qian W, Fang X, Shi Z, Li Y, Li S, Shan G, Kristiansen K *et al*: **De novo assembly of human genomes with massively parallel short read sequencing**. *Genome research* 2010, **20**(2):265-272.
9. **The Caribbean Genome Center Genomes Database** [http://genomes.uprm.edu/parrots]
10. Oleksyk, TK; Guiblet, W; Pombert, JF; Valentin, R; Martinez-Cruzado, JC (2012): Genomic data of the Puerto Rican Parrot (Amazona vittata) from a locally funded project. *GigaScience*. <http://dx.doi.org/10.5524/100039>
11. Ye L, Hillier L, Minx P, Thane N, Locke D, Martin J, Chen L, Mitreva M, Miller J, Haub K et al: **A vertebrate case study of the quality of assemblies derived from next-generation sequences**. Genome Biology 2011, 12(3):R31.
12. Benson DA, Karsch-Mizrachi I, Clark K, Lipman DJ, Ostell J, Sayers EW: **GenBank**. Nucleic Acids Res 2012, 40(Database issue):D48-53
13. Edgar RC: **MUSCLE: a multiple sequence alignment method with reduced time and space complexity**. BMC Bioinformatics 2004, 5:113.
14. Edgar RC: **MUSCLE: multiple sequence alignment with high accuracy and high throughput**. Nucleic Acids Res 2004, 32(5):1792-1797.
15. Drummond A.J., Ashton B., Buxton S., Cheung M., Cooper A., Duran C., Field M., Heled J., Kearse M., Markowitz S. et al: **Geneious v5.5.** In. http://www.geneious.com; 2010.
16. Altschul SF, Gish W, Miller W, Myers EW, Lipman DJ: **Basic local alignment search tool.** J Mol Biol 1990, 215(3):403-410.
17. Smit AFA, Hubley R, Green P: **RepeatMasker Open-3.0**. In.: http://www.repeatmasker.org; 1996-2010.
18. Bhagwat M, Young L, Robison RR: **Using BLAT to find sequence similarity in closely related genomes**. Curr Protoc Bioinformatics 2012, Chapter 10:Unit10 18.
19. International Chicken Genome Sequencing C: **Sequence and comparative analysis of the chicken genome provide unique perspectives on vertebrate evolution.** Nature 2004, 432(7018):695-716.
20. Warren WC, Clayton DF, Ellegren H, Arnold AP, Hillier LW, Kunstner A, Searle S, White S, Vilella AJ, Fairley S et al: **The genome of a songbird.** Nature 2010, 464(7289):757-762.
