# Supplementary material for: A locally funded Puerto Rican parrot (Amazona vittata) genome sequencing project increases avian data and advances young researcher education
Source: Gigascience. 2012 Sep 28;1:14. doi: 10.1186/2047-217X-1-14 (PMC3626513; doi:10.1186/2047-217X-1-14)
Supplement: Additional file 2 — Table S1. Quality and volume of four DNA samples extracted from whole blood of two Amazona vittata parrots selected for the genome sequencing. [file 2047-217X-1-14-S2.doc]

**Table S1 Quality and volume of four DNA samples extracted from whole blood of two *Amazona vittata* parrots selected for the genome sequencing**

| **Sample #** | **Sample name** | **Concentration (ng/ul) picogreen method** | | **Purity (A260/A280)** | | **Volume (ul)** | **Total DNA amount (ug)** |
| --- | --- | --- | --- | --- | --- | --- | --- |
| 1 | Pa1a | 180.68 | 1.79 | | 550 | | 99.37 |
| 2 | Pa9a | 171.15 | 1.79 | | 550 | | 94.13 |
| 3 | Pa15a | 242.57 | 1.87 | | 550 | | 133.41 |
| 4 | Pa16a | 258.89 | 1.87 | | 260 | | 67.31 |
