# Supplementary material for: A locally funded Puerto Rican parrot (Amazona vittata) genome sequencing project increases avian data and advances young researcher education
Source: Gigascience. 2012 Sep 28;1:14. doi: 10.1186/2047-217X-1-14 (PMC3626513; doi:10.1186/2047-217X-1-14)
Supplement: Additional file 3 — Table S2. Results of the genome sequencing (Illumina HiSeq, Axeq Technologies). Pa9a_1 and Pa9a_2 represent the opposite ends of the 300 bp short reads, and the Pa9a-MP_1 and Pa9a-MP_2 are the 2,500 bp mate pairs (MP). All sequences were 101 bp long. [file 2047-217X-1-14-S3.doc]

**Table S2.** Results of the genome sequencing (Illumina HiSeq, Axeq Technologies). Pa9a_1 and Pa9a_2 represent the opposite ends of the 300 bp short reads, and the Pa9a-MP_1 and Pa9a-MP_2 are the 2,500 bp mate pairs (MP). All sequences were 101 bp long.

| **Sequence name** | **Bases** | | | | |
| --- | --- | --- | --- | --- | --- |
|  | **A** | **C** | **G** | **T** | **N** |
| Pa9a_1 | 3,848,744,755 | 2,897,323,917 | 2,900,927,647 | 3,825,967,145 | 23,781,474 |
| Pa9a_2 | 3,868,006,366 | 2,890,106,491 | 2,911,110,520 | 3,826,704,806 | 816,755 |
| Pa9a-MP_1 | 2,174,635,550 | 1,720,812,735 | 1,688,621,488 | 2,158,688,549 | 246,593 |
| Pa9a-MP_2 | 2,171,866,496 | 1,674,935,595 | 1,748,437,549 | 2,147,171,234 | 594,041 |
