# Supplementary material for: A locally funded Puerto Rican parrot (Amazona vittata) genome sequencing project increases avian data and advances young researcher education
Source: Gigascience. 2012 Sep 28;1:14. doi: 10.1186/2047-217X-1-14 (PMC3626513; doi:10.1186/2047-217X-1-14)
Supplement: Additional file 4 — Table S3. Results of the genome assembly by SOAPdenovo [8]. [file 2047-217X-1-14-S4.doc]

**Table S3.** Results of the genome assembly by SOAPdenovo [8]

|  | **Statistics Category** | **paired-end (27.0Gb)** | **PE+ MP 5Gb** | **PE+ MP All** |
| --- | --- | --- | --- | --- |
|  |  |  | (27.0Gb + 5.2Gb) | (27.0Gb + 15.5Gb) |
| **All**  **Contigs** | # Contigs | 12,764,879 | 12,887,828 | 12,887,828 |
|  | Total Length | 1,560,663,735 | 1,772,155,983 | 1,772,155,983 |
|  | Largest Contig | 15,182 | 18,359 | 18,359 |
|  | Mean Length | 122.3 | 137.5 | 137.5 |
|  | N50 | 673 | 636 | 636 |
| **Of All,**  **Contigs**  **≥ 100bp** | # Contigs | 1,884,625 | 4,450,396 | 4,450,396 |
|  | Total Length | 1,099,661,342 | 1,396,126,299 | 1,396,126,299 |
|  | Mean Length | 583.5 | 313.7 | 313.7 |
|  | N50 | 1,188 | 1,123 | 1,123 |
| **Scaffolds** | Largest Scaffold | N/A | 2,014,591 | 3,309,686 |
|  | # Scaffolds | N/A | 3,410,722 | 3,384,799 |
|  | Total Length | N/A | 1,530,900,674 | 1,590,552,602 |
|  | Mean Length | N/A | 448.8 | 470 |
|  | N50 | N/A | 74,348 | 126,952 |

These results are not used in the study, but have been deposited at <http://genomes.uprm.edu/parrot/Assembly-2011/>
