# Supplementary figures and images for: A locally funded Puerto Rican parrot (Amazona vittata) genome sequencing project increases avian data and advances young researcher education
Source: Gigascience. 2012 Sep 28;1:14. doi: 10.1186/2047-217X-1-14 (PMC3626513; doi:10.1186/2047-217X-1-14)

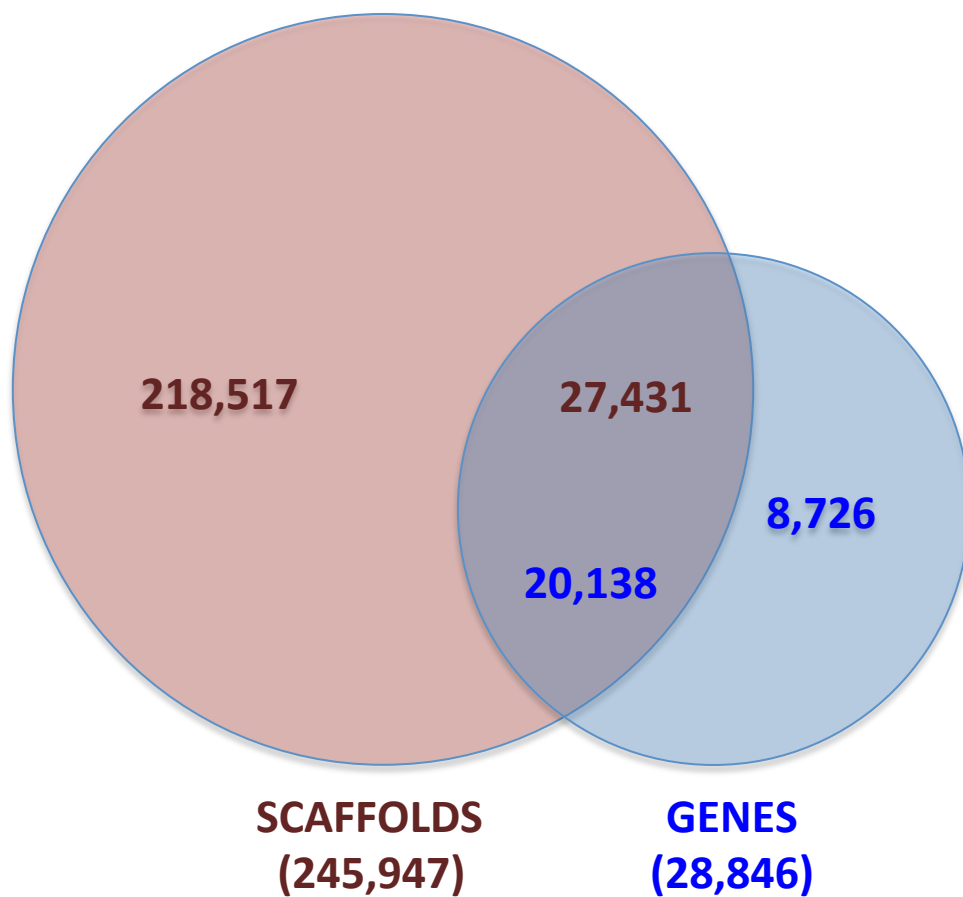

Figure S1

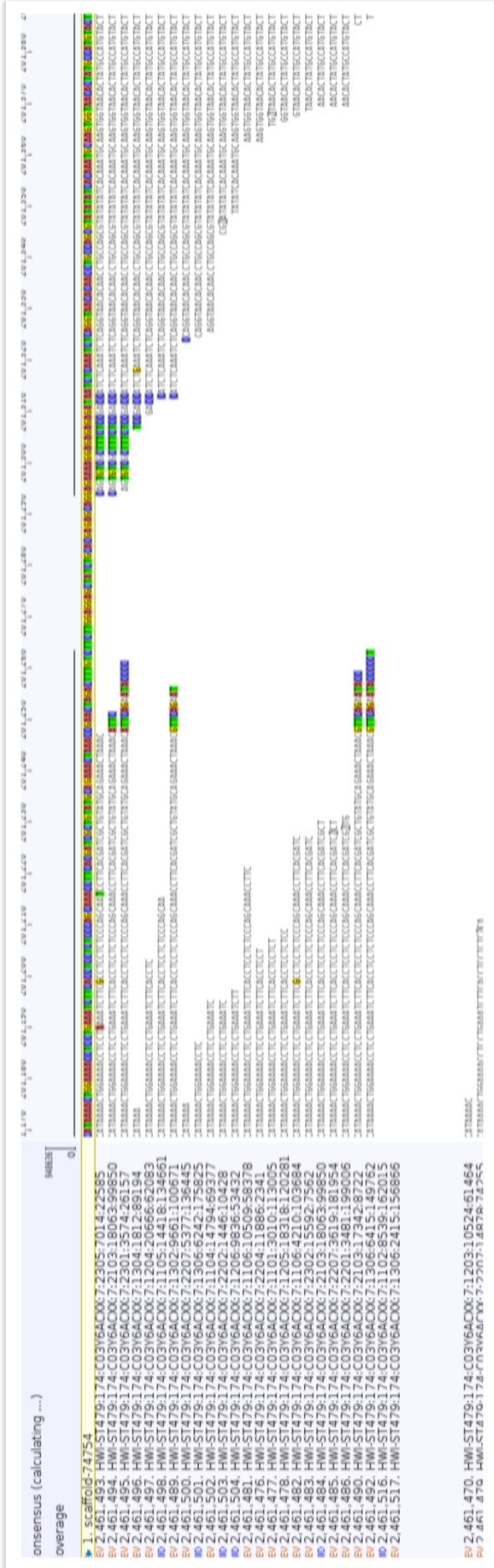

# Figure S2

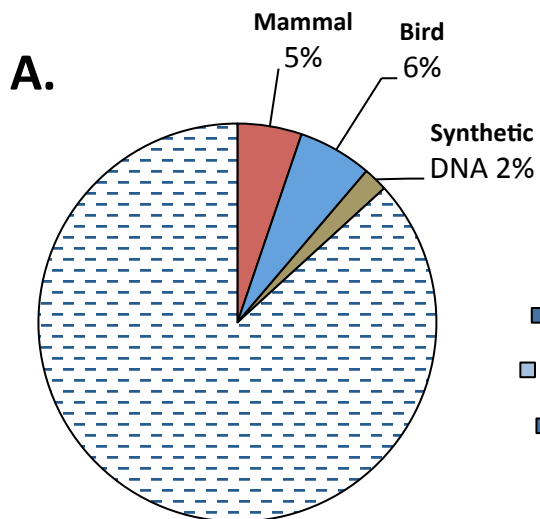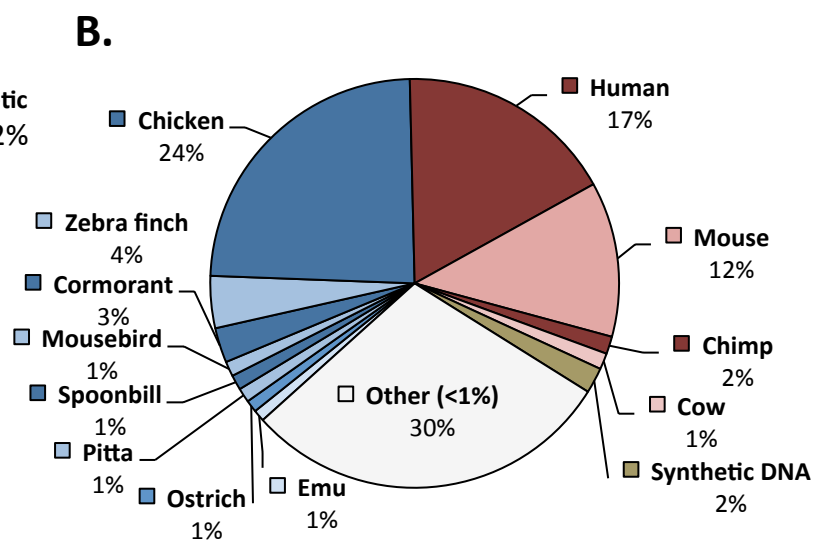

Figure S3

**A.**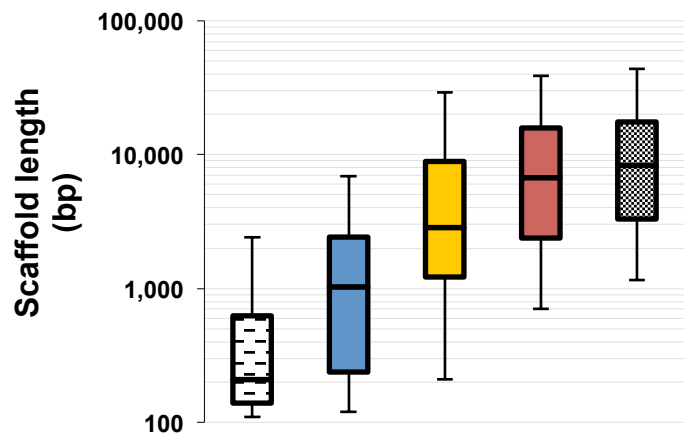**B.**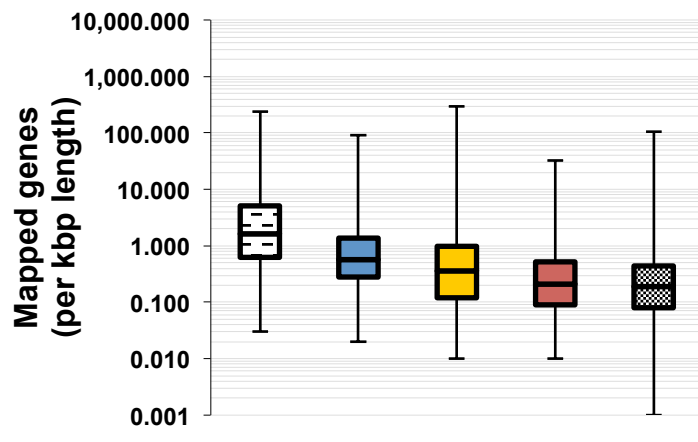**C.**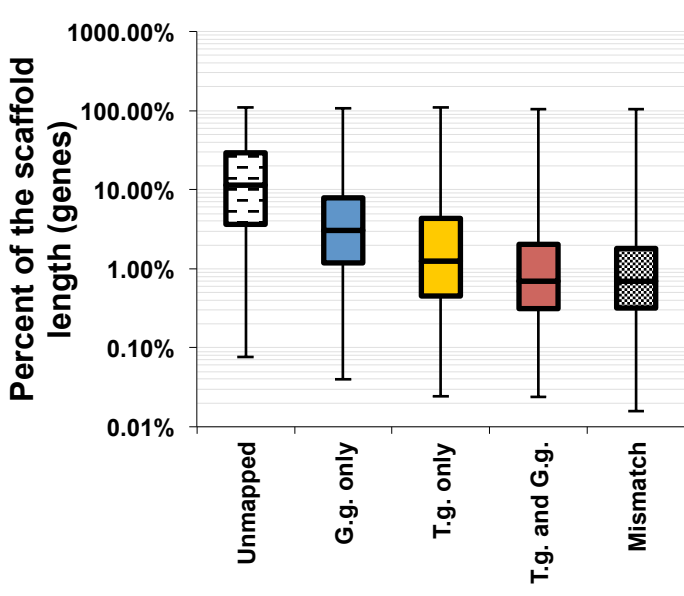**D.**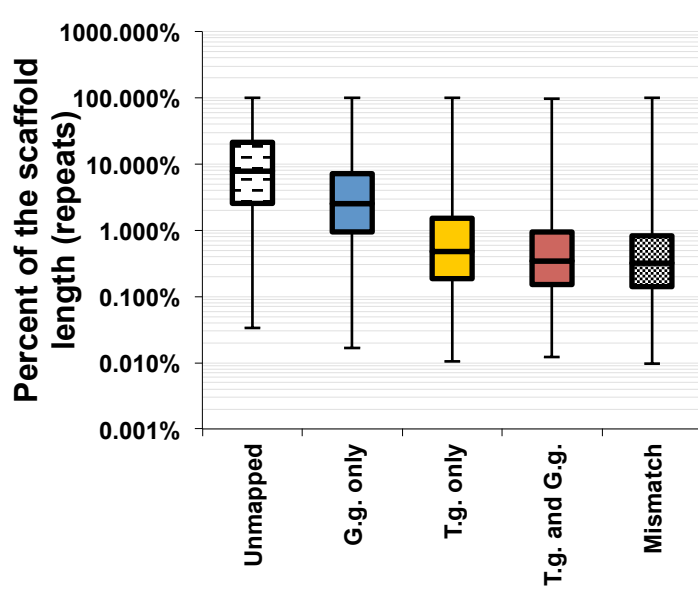

Figure S4

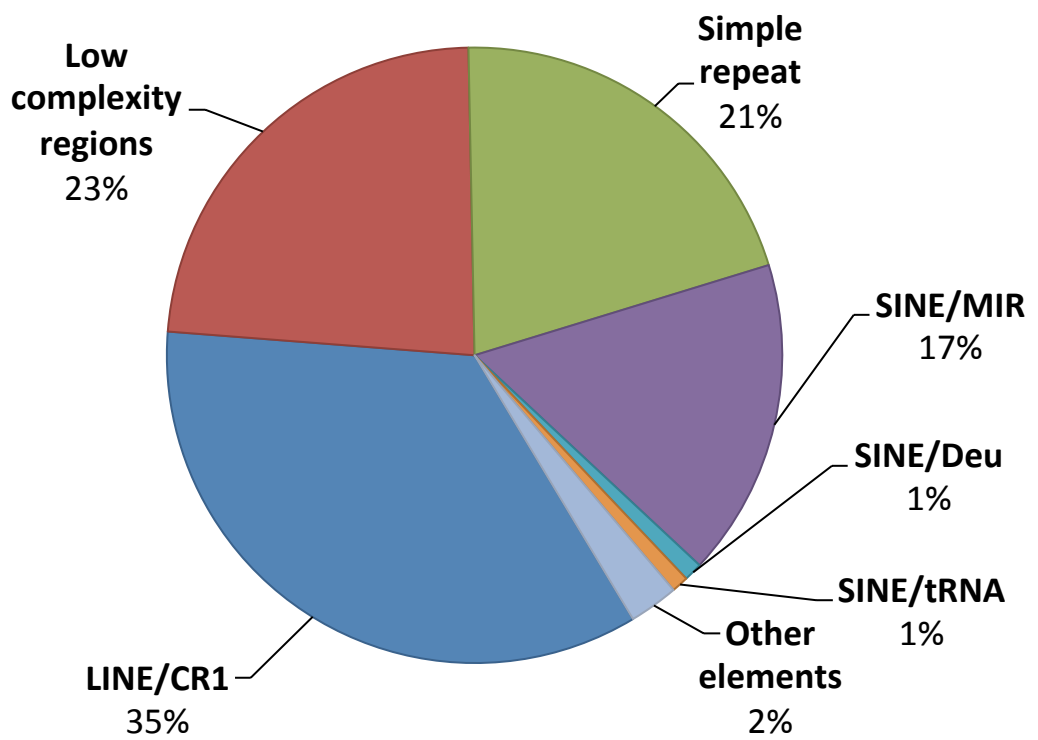

Figure S5

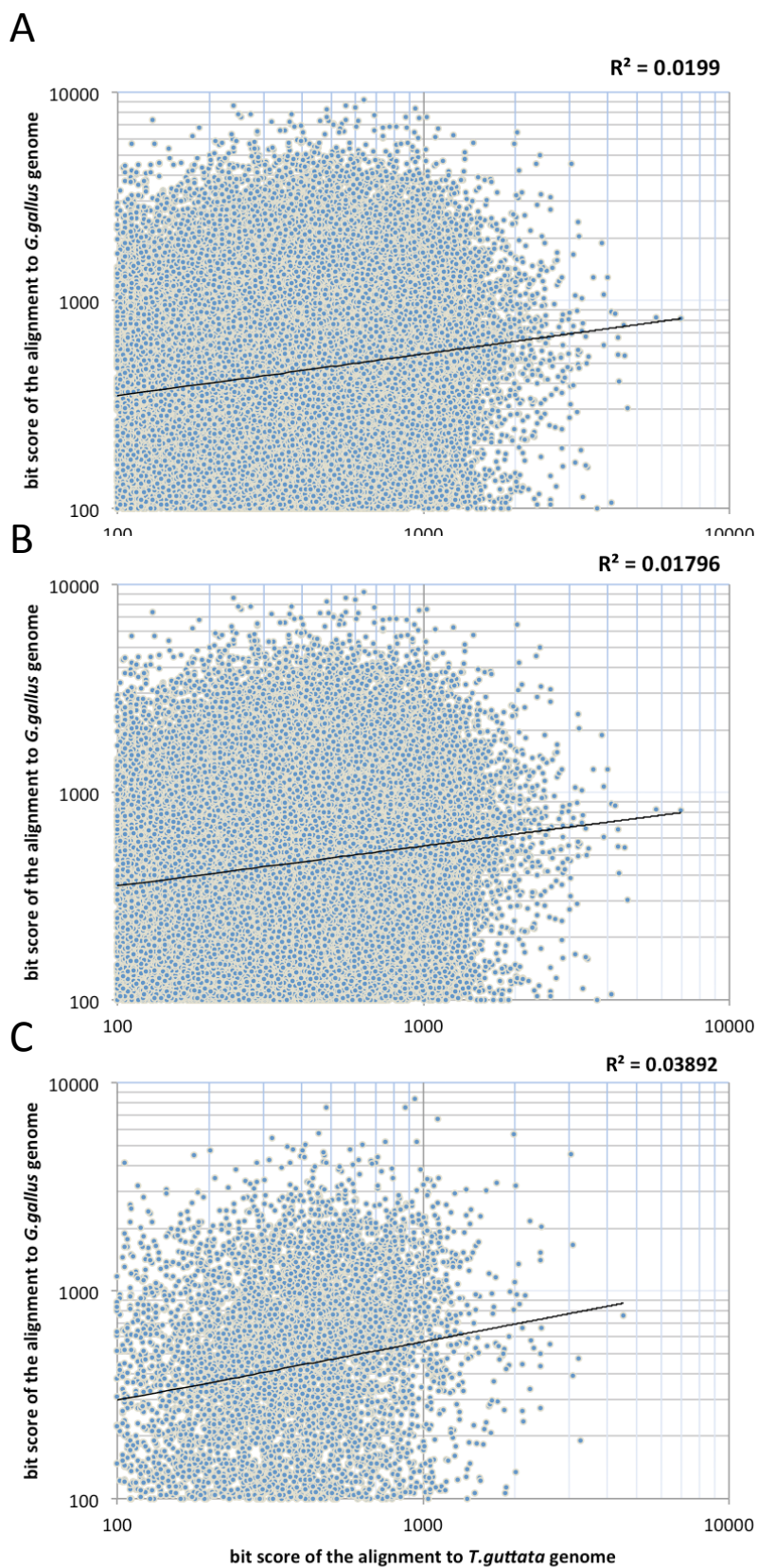

Figure S6

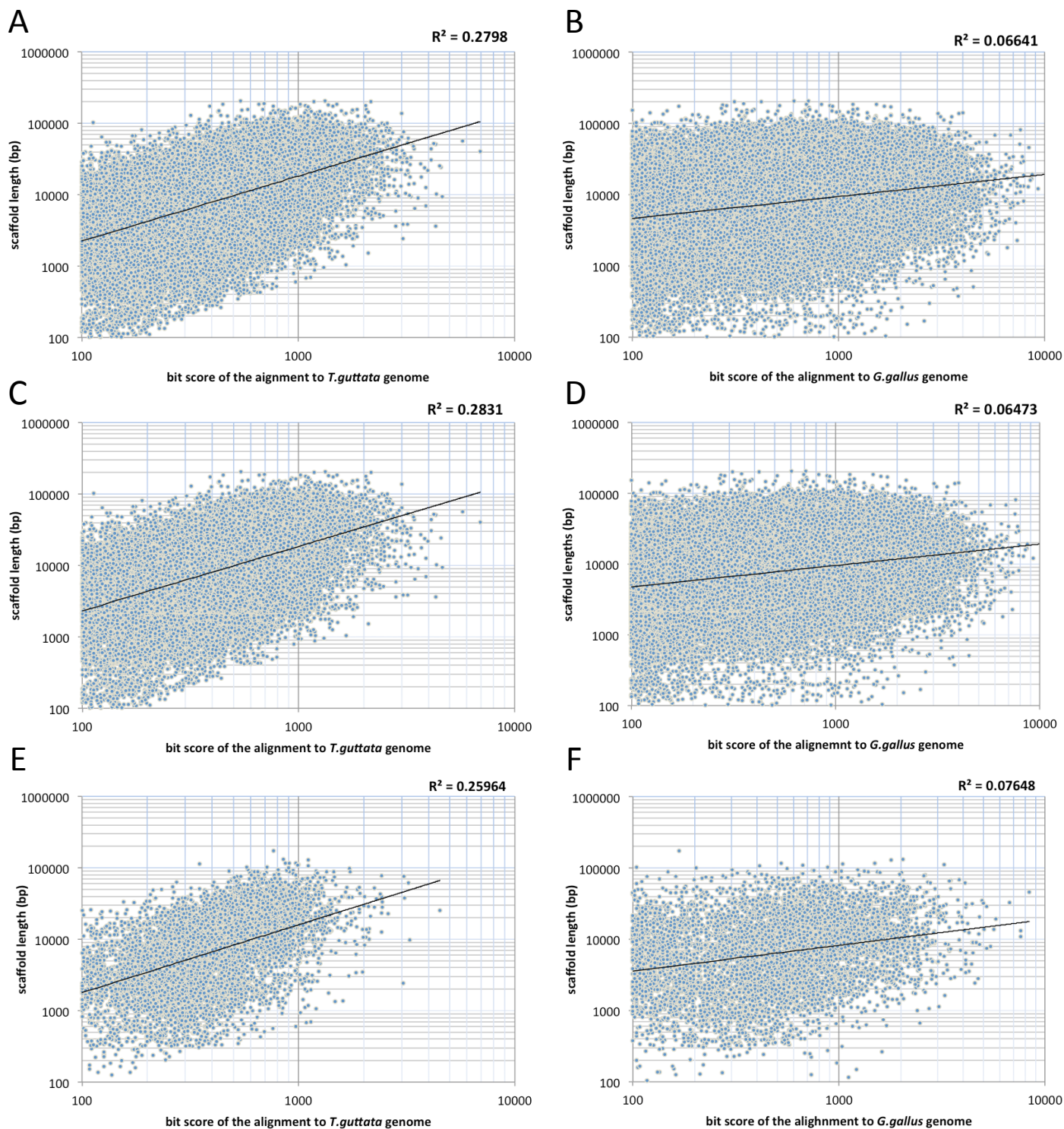

Figure S7

A.

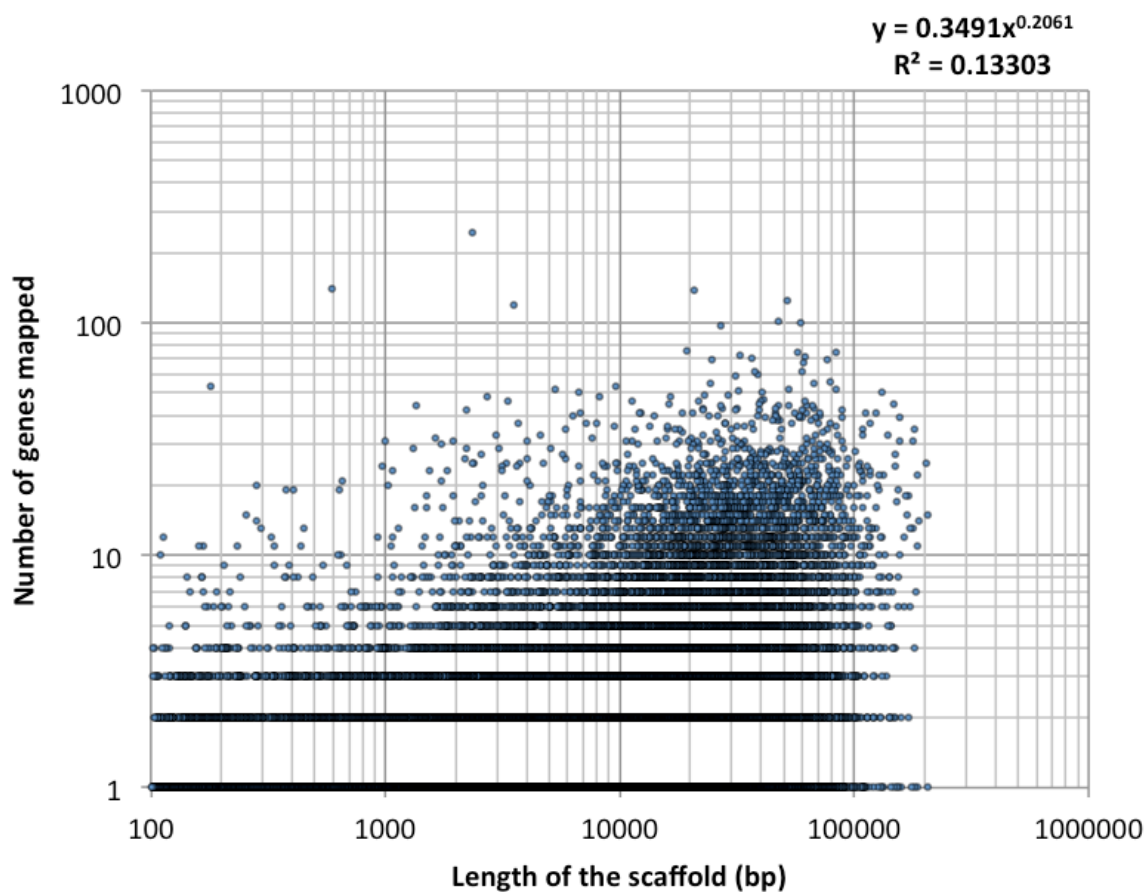

B.

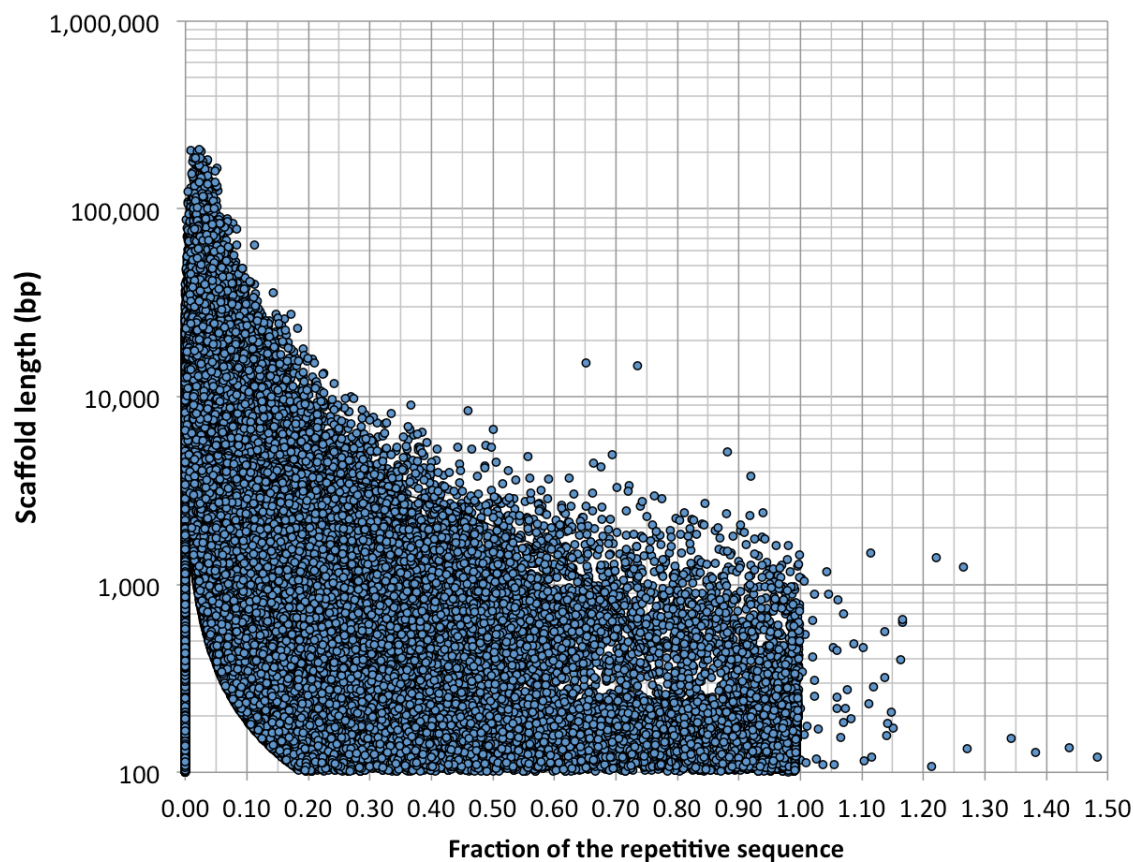

Figure S8

Supplement: Additional file 5 — Supplementary figures. Figure S1. Venn diagram of the overlap between the number of A. vittata scaffolds and the G. gallus transcripts from GenBank that were mapped to them by BLAST. Figure S2. A single example of chimera detected on scaffold-74754 after visual inspection of reads mapped to 100 largest scaffolds. Figure S3. Percentage of scaffolds containing fragments with > 95% similarity to GenBank sequences. Figure S4. Comparison between categories of A. guttata scaffolds (described earlier in Figure 2): The box plots show the medians, Q1, Q3 and the extreme values. The means are shown in Table 3. A. Distribution of scaffold lengths; B. Distribution of densities of genes mapped per kbp of scaffold length. C. Differences in the distribution of proportion of the length of the scaffold mapped to a G. gallus transcript from NCBI Entrez Gene database. D. Differences in the distribution of proportion of the length of the scaffold mapped to a known repeat class using RepeatMasker software [5]. Figure S5. Distribution of major classes of repetitive sequences found on A. vittata scaffolds. Figure S6. Relationship between the quality scores of the alignments between the parrot scaffolds to the chicken and zebra finch genomes: A. All scaffolds. B. Mismatched scaffolds only (those scaffolds that shared similarity with sequences of G. gallus and T. guttata genomes but mapped to different chromosomes in the two species; see classification in Figure 2). C. Matched sequences only (those that mapped to the same chromosome in reference genomes of the two avian species). Figure S7. Relationship between the size of a scaffold and the quality of its alignment to T. guttata and/or G. gallus genome sequence: A. All scaffolds aligned to the T. guttata genome. B. All scaffolds aligned to the G. gallus genome. C. Scaffolds from T. guttata that Mismatched scaffolds mapped to different chromosomes in G. gallus; see classification in Figure 2). D. Scaffolds from G. gallus that Mismatched sca [file 2047-217X-1-14-S5.pdf]
