# Supplementary material for: A locally funded Puerto Rican parrot (Amazona vittata) genome sequencing project increases avian data and advances young researcher education
Source: Gigascience. 2012 Sep 28;1:14. doi: 10.1186/2047-217X-1-14 (PMC3626513; doi:10.1186/2047-217X-1-14)
Supplement: Additional file 15 — Table S9. Bioinformatics tools and outputs for scaffold and gene annotation. [file 2047-217X-1-14-S15.doc]

**Table S9.** Bioinformatics tools and outputs for scaffold and gene annotation

| **Elements Annotated** | **Tools** | **Outputs per Scaffold or Gene** |
| --- | --- | --- |
| **Scaffolds** | BLAT  n-BLAST  Homologene  Gene,  UniProtKB  RepeatMasker | - Chromosome number and end coordinates of BLAT matches  - Match scores  - # of elements conserved  - # and identity of RefSeq genes  - Gene orthology and ontology |
| **Genes** | UniProtKB  Ensembl  x-BLAST  UCSC Genome Browser | - Coordinates for start and end of each coding region plus splice sites within |

**Table S10.** An example of annotation output produced by a student in the Genome annotation class using *A. vittata* genome
